# Supplementary figures and images for: Genetic variation influences food-sharing sociability in honey bees
Source: PLoS Biol. 2025 Sep 16;23(9):e3003367. doi: 10.1371/journal.pbio.3003367 (PMC12440162; doi:10.1371/journal.pbio.3003367)

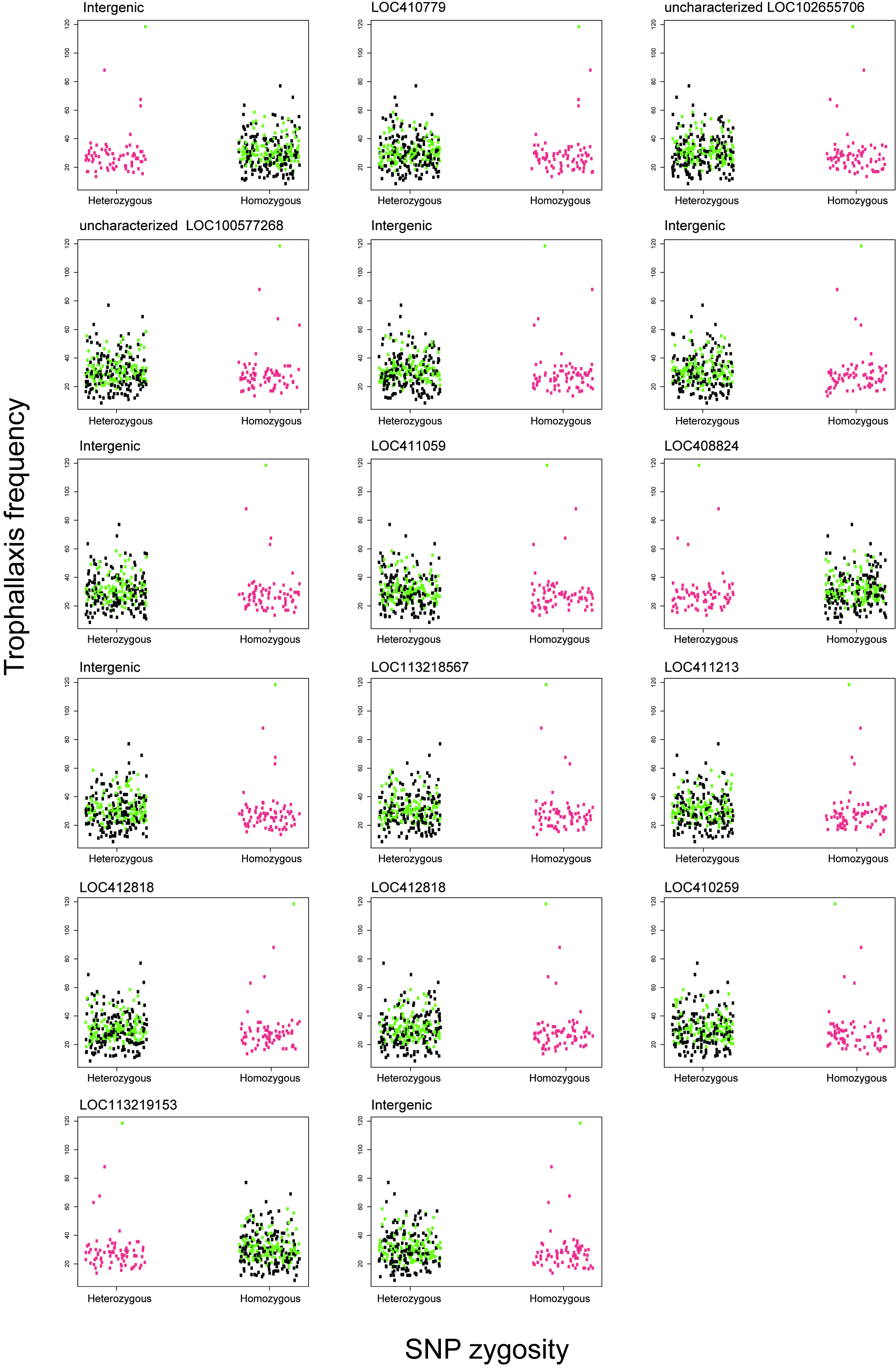

Supplement: S1 Fig — All SNPs are described in S1 Table; 17 out of 18 SNPs are shown, with the remaining SNP shown in Fig 2a. Gene identifiers are listed for intronic SNPs. Code and data underlying S1 Fig can be found at https://doi.org/10.6084/m9.figshare.29845490. (TIF) [file pbio.3003367.s001.tif]

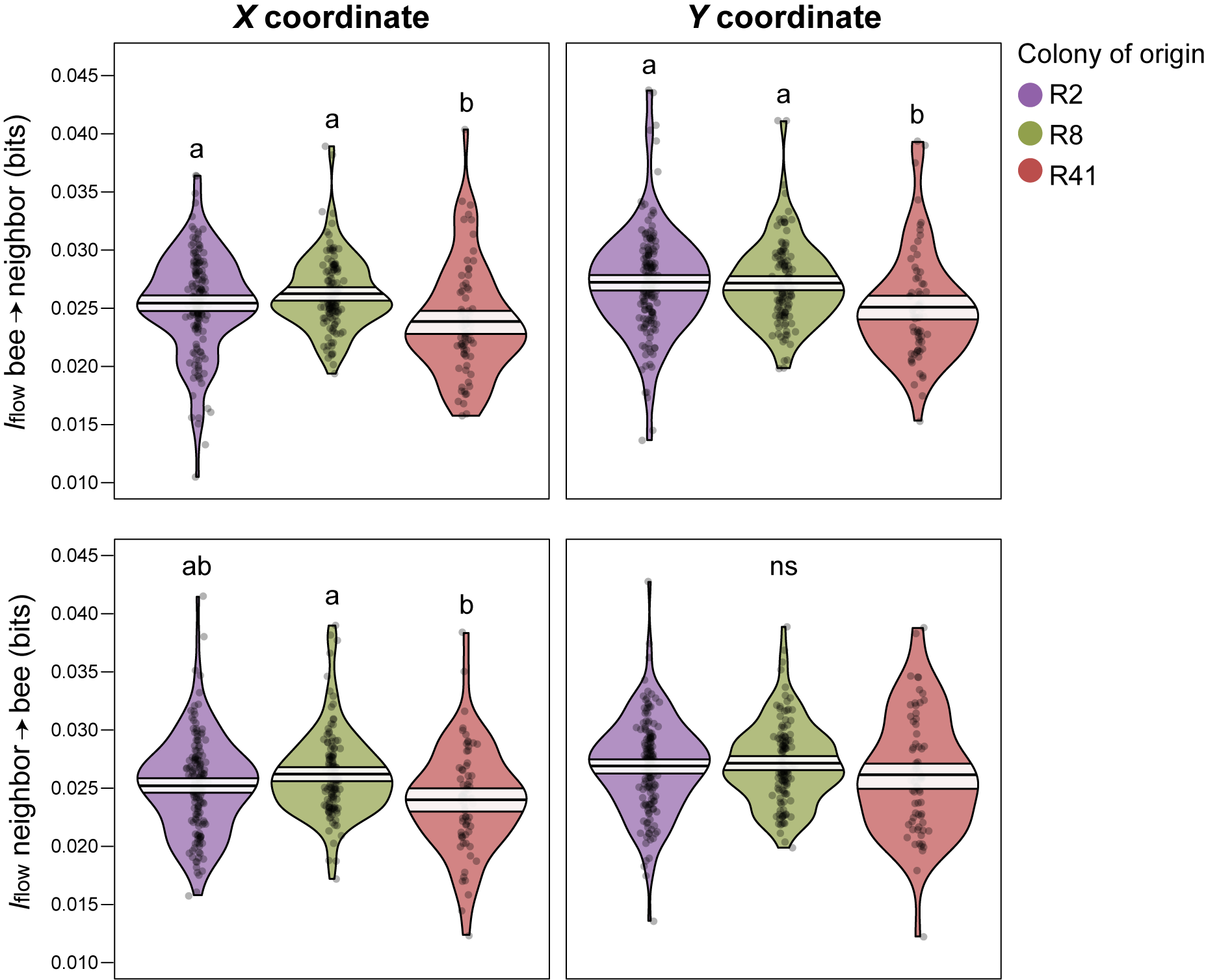

Supplement: S2 Fig — Iflow was then averaged across the two coordinates obtaining a single Iflow for each bee (Fig 2h). Violin plots are constructed as follows: points represent raw data, solid black lines represent the mean, pale white shading above and below the mean represent a 95% confidence interval, and plot shape represents a smoothed density curve outlining the distribution of raw data. Letters above violin plots represent significance (P-value < 0.05) from a between-group Tukey post-hoc analysis following a one-way ANOVA (for X coordinate Iflow bee->neighbor: F(2,352) = 8.03, P = 3.87e-04, Iflow neighbor->bee: F(2,352) = 7.02, P = 0.001); for Y coordinate Iflow bee->neighbor: F(2,352) = 6.92, P = 0.001, Iflow neighbor->bee: F(2,352) = 1.4, P = 0.248). Code and data underlying S2 Fig can be found at https://doi.org/10.6084/m9.figshare.29845490. (TIF) [file pbio.3003367.s002.tif]

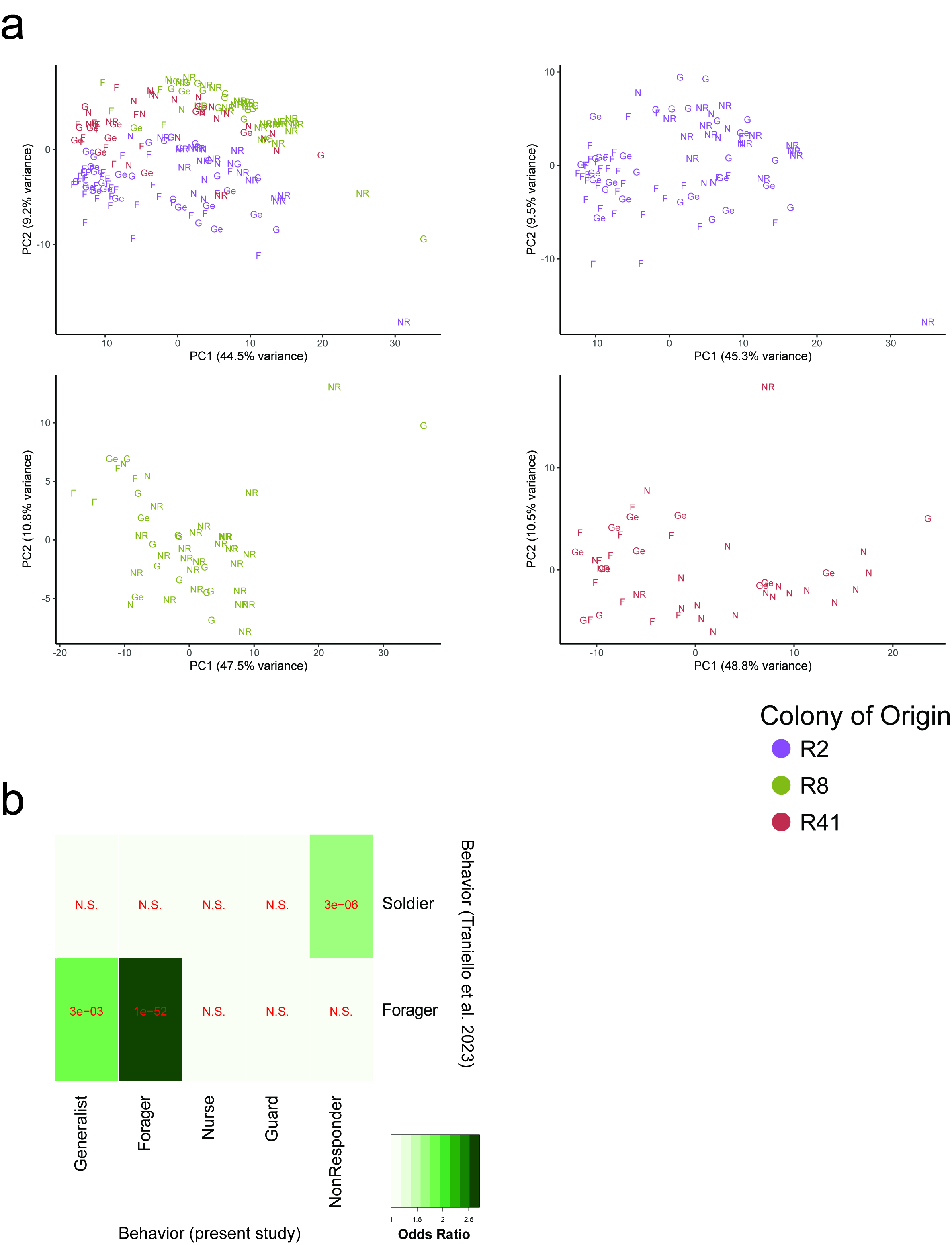

Supplement: S3 Fig — Each letter represents an individual bee assigned to a specific behavioral group based on automated observations of foraging and manual observations of behavior in a lab-based dish assay, as described in Methods. Behavioral groups are denoted as follows: Ge, Generalist; F, Forager; N, Nurse; G, Guard; NR, NonResponder. (b) Gene list overlap heatmap comparing mushroom body transcriptomic profiles of Generalists, Foragers, Nurses, Guards, and NonResponders in the present study to whole-brain transcriptomic profiles of Soldiers and Foragers from Traniello and colleagues (2023). Odds ratio is calculated via hypergeometric overlap test and resulting P-values are reported following correction with the Bonferroni method; N.S., non-significant. Code and data underlying S3 Fig can be found in S2-S8 Tables and https://doi.org/10.6084/m9.figshare.29845490. (TIF) [file pbio.3003367.s003.tif]
