# Supplementary material for: Genetic variation influences food-sharing sociability in honey bees
Source: PLoS Biol. 2025 Sep 16;23(9):e3003367. doi: 10.1371/journal.pbio.3003367 (PMC12440162; doi:10.1371/journal.pbio.3003367)
Supplement: Attachment — Submitted filename: PLOS_Review.docx [file pbio.3003367.s013.docx]

I enjoyed reading this manuscript and found the results exciting. At the same time, the different subsections of the results felt disjointed and I was confused by aspects of the overall framing.

The authors use a GWAS to show that 18 SNPs correlate with the frequency at which honeybees engage in trophallaxis. Two of these SNPs are in the gene *neurolignin-2* (*nlg-2*), which is a focus of the paper.

In a following section, the authors show that bees that trophallax more frequently have a stronger influence on the movement of neighboring bees (‘social influence’) than bees that trophallax less frequently. This data is presented at the colony-level, not the individual-level even though the individual-level data exists. There could be an equivalent GWAS for these individual-level data, as was done for trophallaxis. My sense is that it might even be more informative, for reasons that are provided below. In the current form this results subsection feels disconnected.

The authors next look at gene expression in the mushroom body and find that 1705 genes correlate with trophallaxis frequency. It is not stated whether *nlg-2* is among these. Instead, a separate analysis is performed to test whether *nlg-2* expression correlates with trophallaxis frequency and behavioural role in the colony. The data on behavioral role is based on a suite of assays and measures, the data from which seems to be used only here, and even here only in passing, which is a shame because they are interesting.

In a last results section, the authors use FISH to localize the expression of *nlg-2* to neuronal populations that are dispersed throughout the brain and show that expression is high in bees that are one-day old, negligible in bees that are one-week old, and elevated again in bees that are two-weeks old. This pattern is odd but is not discussed further. It is also not clear why the authors focus on age here, rather than sociability. It is the first time age is discussed.

My concerns are:

1) The authors use trophallaxis as a measure of ‘sociability’. They control for movement kinetics, but not task behavior. I believe trophallaxis frequency varies with behavioural role (this is even written on L. 85). Do nurses and foragers trophallax at the same rate? I know there is evidence in ants that certain individuals specialize in food dissemination. I do not think these are more ‘social’. It would be important to see how trophallaxis frequency relates to the performance of different tasks. The authors have the data. This concern would also be allayed if the authors have data showing that trophallaxis frequency does not vary with age. Related to this point:

- L.82: “In addition to task-related behaviors, honey bees frequently engage in mouth-to-mouth sharing of liquid…” I would disagree with this framing, and rather see trophallaxis as task-related.
- L. 90: “Taken together, trophallaxis is a quantifiable analog to other measures of sociability across diverse taxa”. I would like to see more evidence of how trophallaxis varies for individuals over time, in association with task performance, and with other measures of sociability to justify this claim.
- L. 107: “we quantified movement kinetics to disentangle trophallaxis engagement frequency from overall activity rate”. This is a great control, but the missing control of task performance is an issue.

2) The connection between the results subsections generally seem weak. The social influence analysis is tied in tangentially, and at a colony level while the trophallaxis analysis is at the individual level. The analysis of task behavior is almost not used, and the FISH data quantifies age-associated differences rather than differences in task behavior or trophallaxis frequency.
3) In the discussion there are a few points where the authors make evolutionary claims that I believe are unfounded:

- L. 500: Is there evidence for the idea that ‘trophallaxis relationships rely on reciprocity between nestmates’? I expect the behavioural dynamics of social insect societies are shaped entirely by inclusive fitness effects, and not at all by reciprocity.
- L. 550: “Basic evolutionary theory supports the speculation that socially divergent phenotypes are either tolerated or preserved across diverse animal lineages due to supportive, or perhaps even vital, roles they contribute to their society”. I do not believe that basic evolutionary theory supports that notion! It does not contradict it either, but there are a whole host of possible explanations for the presence of socially divergent phenotypes. For example, socially divergent phenotypes could be maladaptive but persist if the rate of developmental error is low and the cost of reducing the error rate further is high. Which is likely the reality.

Some minor additional points:

L. 80: “tracking now allows for behavioural monitoring… over long portions of [social insects] lives”: There are ant papers which do exactly that, but the cited example is not one of them.

L. 151: Trophallaxis events are identified with a CNN. It would be useful to quantify the accuracy of this by comparing with manual observations.

L. 226: The automated detection of foragers is great. And the other behavioural assays. This data should be controlled for when performing GWAS on trophallaxis frequency.

L. 433: “Non-responders were transcriptomically distinct from generalists, foragers, guards, and nurses”. To assess this, it would be useful to see the RNA-Seq data plotted out on a PCA with individuals from these different groups highlighted.

L. 467: This temporal pattern is strange. Does it relate to behaviors that old and young bees share and that middle-aged bees do not exhibit?

L. 453-454: Part of this sentence is missing: “when (“responders,” Wilcoxon…”

L. 450: The link between this sentence and the next is confusing. The first states that the authors are testing whether *nlg* expression relates to task performance. The next sentence states that expression levels were correlated with ‘trophallaxis sociability’, without providing the result from the test that the first sentence stated was being done!

L. 508: It would be useful to have a more detailed explanation of which specific *Drosophila* social behaviors are affected by mutations in *nlg-2*.
