# Supplementary material for: Genetic variation influences food-sharing sociability in honey bees
Source: PLoS Biol. 2025 Sep 16;23(9):e3003367. doi: 10.1371/journal.pbio.3003367 (PMC12440162; doi:10.1371/journal.pbio.3003367)
Supplement: Attachment — Submitted filename: PlosBiol_ResponseToReviewers_1July2025.docx [file pbio.3003367.s015.docx]

We thank the four Reviewers for the excellent feedback that improved the manuscript. As a general note, line-numbers in this document, which correspond to changes made in the manuscript, are referencing the “track-changes” version of the manuscript. A clean version has also been uploaded.

Reviewer #1:
[see also the fully formatted version in the attachment]

I enjoyed reading this manuscript and found the results exciting. At the same time, the different subsections of the results felt disjointed and I was confused by aspects of the overall framing.

We thank the Reviewer for the positive feedback and hope our revisions, as discussed below, improve continuity for the reader.

The authors use a GWAS to show that 18 SNPs correlate with the frequency at which honeybees engage in trophallaxis. Two of these SNPs are in the gene neurolignin-2 (nlg-2), which is a focus of the paper.

In a following section, the authors show that bees that trophallax more frequently have a stronger influence on the movement of neighboring bees ('social influence') than bees that trophallax less frequently. This data is presented at the colony-level, not the individual-level even though the individual-level data exists. There could be an equivalent GWAS for these individual-level data, as was done for trophallaxis. My sense is that it might even be more informative, for reasons that are provided below. In the current form this results subsection feels disconnected.

This is an excellent idea for connecting the disjointed sections. We have two updates to the manuscript based on this suggestion. First, we performed a new GWAS using the social influence data (both “bee->neighbor” and “neighbor->bee,” following the terminology used in Figure 3d-h), as described on Lines 347-352. Next, we calculated a polygenic risk score (PRS, described in Lines 351-352) associated with the 18 trophallaxis sociability-associated SNPs and regressed the social influence data on this new metric. While we did not identify any genetic variation associated with either influence metric, we found a significant, negative relationship between PRS and both social influence metrics, an exciting finding we report in Lines 489-492 of the Results and discuss in Lines 652-658 of the Discussion.

We thank the Reviewer for this suggestion, which has allowed us to make a new and important connection across analyses.

The authors next look at gene expression in the mushroom body and find that 1705 genes correlate with trophallaxis frequency. It is not stated whether nlg-2 is among these. Instead, a separate analysis is performed to test whether nlg-2 expression correlates with trophallaxis frequency and behavioural role in the colony. The data on behavioral role is based on a suite of assays and measures, the data from which seems to be used only here, and even here only in passing, which is a shame because they are interesting.

We agree that the mushroom body transcriptomic data are interesting! In fact we initially developed an in-depth transcriptomic analysis that uncovered unexpected links between division of labor, brain transcriptomic state, and the “non-responder” honey bee phenotype, which nicely integrated with other parts of this manuscript. Specifically, we found that brain gene expression in “non-responders” in this study closely matched that of “soldier” honey bees, in another of our studies. Soldiers are a small subset of the colony defensive force that fly out of the hive to bite and sting intruders. Because the “non-responder” phenotype was previously connected to human autism in Shpigler et al. (2017), we were intrigued by these results. But given the current polarization around the autism discourse, we chose to keep this material out of the manuscript that we submitted.

This Reviewer’s comment, however, has inspired us to add these analyses with a new framing that uses insights from the non-responder phenotype to suggest that decreased social responsiveness may be necessary in the context of division of labor, as a soldier’s dedicated attention to monitoring and removing threats may be worth the decrease of social interactivity. We now speculate that “non-responders” may also show decreased attention to social stimuli but excel in a specific task that requires extreme attention and precision. This is a documented phenomenon in autism, sometimes termed “circumscribed” interest, in which an individual on the spectrum demonstrates extraordinary ability in a specific task domain despite social proclivities that deviate from a population-level norm. We now present these findings and speculation in Supplementary Figure 3b, Lines 514-525 of the Results and 689-699 of the Discussion.

In a last results section, the authors use FISH to localize the expression of nlg-2 to neuronal populations that are dispersed throughout the brain and show that expression is high in bees that are one-day old, negligible in bees that are one-week old, and elevated again in bees that are two-weeks old. This pattern is odd but is not discussed further. It is also not clear why the authors focus on age here, rather than sociability. It is the first time age is

We agree that this section needs better description if it is to be properly integrated into the rest of the manuscript. We have now supplied a more coherent discussion of behavioral maturation that clarifies the significance of the FISH collection times. This new text can be found in Lines 56-61 of the Introduction (also in response to the first concern below), in the Methods (Lines 386-389), Results (551-556), and a new paragraph in the Discussion, Lines 663-677.

In brief, our effort in collections for FISH was to understand how *nlg2* expression and distribution changes with behavioral maturation, which is intrinsically linked to division of labor (specifically, the hive bee-to-forager transition). Indeed, the pattern is odd! But we now supply several references regarding neuroanatomical observations that track the hive bee-to-forager transition and more carefully unpack these results to tie them back to the bigger picture. We thank the Reviewer for the comment as we feel this improves the presentation of the FISH results.

My concerns are:

1)      The authors use trophallaxis as a measure of 'sociability'. They control for movement kinetics, but not task behavior. I believe trophallaxis frequency varies with behavioural role (this is even written on L. 85). Do nurses and foragers trophallax at the same rate? I know there is evidence in ants that certain individuals specialize in food dissemination. I do not think these are more 'social'. It would be important to see how trophallaxis frequency relates to the performance of different tasks. The authors have the data. This concern would also be allayed if the authors have data showing that trophallaxis frequency does not vary with age. Related to this point:

This is an excellent point, and we see there is need for us to clarify this analysis in light of a recent publication where we explored the relationship between task allocation and trophallaxis frequency (Traniello et al. 2022, JEB). This previous study contains a much larger behavioral dataset, including five colony replicates and thousands of bees, and helped shape the conceptualization of the sociability GWAS in the present manuscript. In brief, previous results tell us that trophallaxis and behavioral state (i.e., nurse, guard, forager, generalist, non-responder) are significantly correlated: same-aged bees that regularly perform different tasks in the hive also engage in trophallaxis at different rates, with generalists/foragers engaging the most and non-responders engaging the least. With knowledge of this robust relationship, we knew that “controlling” for behavior (by, for example, including behavioral state as a term in the GWAS model) would violate collinearity assumptions.

We now have made this point explicit in the Methods in Lines 350-352, and also have substantially modified the Introduction (Lines 56-61) to clarify the relevance of the present study in light of what we know about trophallaxis and behavior in the hive. This is vital background for the reader, and we thank the Reviewer for indicating as much.

-       L.82: "In addition to task-related behaviors, honey bees frequently engage in mouth-to-mouth sharing of liquid…" I would disagree with this framing, and rather see trophallaxis as task-related.

We have modified the Introduction (Lines 93-98) to more substantially discuss the relationship between trophallaxis and task allocation.

-       L. 90: "Taken together, trophallaxis is a quantifiable analog to other measures of sociability across diverse taxa". I would like to see more evidence of how trophallaxis varies for individuals over time, in association with task performance, and with other measures of sociability to justify this claim.

This is an excellent question! We have done much of this analysis and presented it in Traniello et al. 2022. We now more thoroughly discuss these results in the present manuscript’s Introduction (in Lines 93-98, also answering the above question).

-       L. 107: "we quantified movement kinetics to disentangle trophallaxis engagement frequency from overall activity rate". This is a great control, but the missing control of task performance is an issue.

We clarify this issue in Lines 93-98 and 350-352, in-line with the two previous responses.

2)      The connection between the results subsections generally seem weak. The social influence analysis is tied in tangentially, and at a colony level while the trophallaxis analysis is at the individual level. The analysis of task behavior is almost not used, and the FISH data quantifies age-associated differences rather than differences in task behavior or trophallaxis frequency.

As noted above, we now include a more thorough discussion of the relationship between task allocation, age, and trophallaxis (Lines 56-61 and 93-98) and also include a deeper dive into individual-level social influence behaviors via GWAS. As discussed above, we unpack the FISH results in Lines 663-677.

3)      In the discussion there are a few points where the authors make evolutionary claims that I believe are unfounded:
-       L. 500: Is there evidence for the idea that 'trophallaxis relationships rely on reciprocity between nestmates'? I expect the behavioural dynamics of social insect societies are shaped entirely by inclusive fitness effects, and not at all by reciprocity.

Good point. We are using the term “reciprocity” in a more transactional mechanistic way, rather than in the evolutionary context associated with traditional examples of reciprocal food-sharing interactions (e.g., blood meal regurgitation among vampire bats). We do not know enough about the nature of reciprocity in the beehive (which we now openly state), only that these interactions are relevant to intracolony communication and resource allocation. We now clarify this text in Lines 599-601.

-       L. 550: "Basic evolutionary theory supports the speculation that socially divergent phenotypes are either tolerated or preserved across diverse animal lineages due to supportive, or perhaps even vital, roles they contribute to their society". I do not believe that basic evolutionary theory supports that notion! It does not contradict it either, but there are a whole host of possible explanations for the presence of socially divergent phenotypes. For example, socially divergent phenotypes could be maladaptive but persist if the rate of developmental error is low and the cost of reducing the error rate further is high. Which is likely the reality.

Agreed, and we now have modified Discussion in Lines 714-717. We want to avoid a reading of the manuscript that describes autism, or other developmental disorders, as “maladaptive” and we hope we have done this.

Some minor additional points:

L. 80: "tracking now allows for behavioural monitoring… over long portions of [social insects] lives": There are ant papers which do exactly that, but the cited example is not one of them.

Thank you, we left out an important publication, Mersch et al. (2013), which features > 1 month of carpenter ant tracking in its dataset. This has been added.

L. 151: Trophallaxis events are identified with a CNN. It would be useful to quantify the accuracy of this by comparing with manual observations.

This is requested by another Reviewer as well, so there is clear need to reference previous studies that link the automated predictions to manual observations, all utilizing the same tracking technology, either with or without the CNN (Gernat et al. 2020, Gernat 2023, Geffre et al. 2020, Jones et al. 2020). Before the CNN was implemented, exhaustive manual observations were made to ensure detection accuracy (Gernat et al. 2020); these references also regard ground-truthing the CNN with manual observations (Gernat et al. 2023) and consistency in trophallaxis detection rates between the lab (i.e., observation hive) and field (Geffre et al. 2020). We discuss the relevance of this vital previous work in Lines 172-187 of the Methods.

L. 226: The automated detection of foragers is great. And the other behavioural assays. This data should be controlled for when performing GWAS on trophallaxis frequency.

We thank the Reviewer for the kind words – the automated foraging detector is certainly a useful tool! As noted above, we now discuss the relationship between task allocation and trophallaxis.

L. 433: "Non-responders were transcriptomically distinct from generalists, foragers, guards, and nurses". To assess this, it would be useful to see the RNA-Seq data plotted out on a PCA with individuals from these different groups highlighted.

We now present the PCA in Supplementary Figure 3a, where it leads up to the newly presenting gene list overlap analysis. Supporting text is in Lines 366-369 and 514-525.

L. 467: This temporal pattern is strange. Does it relate to behaviors that old and young bees share and that middle-aged bees do not exhibit?

We think so, and we think this is tied to the neurobiology of in-hive (callow) behaviors, out-of-hive (old) behaviors, and the transition point between! But we did not address this well enough in the text. As addressed in a previous comment, we now unpack these results and contextualize them within the framework of behavioral maturation, specifically in Lines 663-677.

L. 453-454: Part of this sentence is missing: "when ("responders," Wilcoxon…"

These lines have been edited to remove the phrase “, when” and the word “responders,” which were included in error. We thank the Reviewer for catching these errors.

L. 450: The link between this sentence and the next is confusing. The first states that the authors are testing whether nlg expression relates to task performance. The next sentence states that expression levels were correlated with 'trophallaxis sociability', without providing the result from the test that the first sentence stated was being done!

We agree – the results following that sentence don’t come until the end, which is very confusing! This paragraph has been re-written to better express our understanding of the relationship between *nlg2* and the phenotypes of interest (Lines 542-550).

L. 508: It would be useful to have a more detailed explanation of which specific Drosophila social behaviors are affected by mutations in nlg-2.

This is a great idea: the literature is rich, and many of the markers we identified via GWAS have been well studied in fruit flies. We now include a deeper dive in the Discussion, in Lines 628-642. Importantly, *nlg2* appears to affect fruit fly social circuitry without impairing movement, just as we report for our bees.

Reviewer #2:

In the manuscript entitled "Molecular analyses of individual variation in honey bee sociability," Traniello et al. combine genome sequencing, brain transcriptomics, and automated behavioral tracking to identify 18 SNPs associated with variation in trophallaxis (which they consider a proxy for "sociability") in honey bee colonies. Several of these SNPs were associated with genes previously linked to sociability in other species. The authors also find genes whose expression covaried with sociability. Overall, this is a timely and interesting study. The manuscript is well written. I have only a few comments which are intended to help he authors to improve the manuscript further.

General comments:

1) The concordant discovery of neuroligin-2 (nlg2) in both genomic screen and transcriptome analysis is obviously cool. In the absence of a detailed and careful phylogenetic analysis of the entire neuroligin gene family I am wondering how confident we can be about the orthology assignments with putative orthologs in Drosophila and humans? I think such an analysis is beyond the scope of the current study, but I certainly hope someone will do this analysis at some point. Interestingly enough, neuroligins have also been implicated in female mate choice across teleost fishes (see, for example, <https://nam12.safelinks.protection.outlook.com/?url=https%3A%2F%2Fdoi.org%2F10.1242%2Fjeb.207324&data=05%7C02%7Cit4770%40princeton.edu%7C6a4bb1af54a14e9a729508dd7772226b%7C2ff601167431425db5af077d7791bda4%7C0%7C0%7C638798054677071690%7CUnknown%7CTWFpbGZsb3d8eyJFbXB0eU1hcGkiOnRydWUsIlYiOiIwLjAuMDAwMCIsIlAiOiJXaW4zMiIsIkFOIjoiTWFpbCIsIldUIjoyfQ%3D%3D%7C0%7C%7C%7C&sdata=hWKk9A%2F%2FUez6zONfE1LBdNHSkwX9y0kBYtNFD3AcfIk%3D&reserved=0>, <https://nam12.safelinks.protection.outlook.com/?url=https%3A%2F%2Fdoi.org%2F10.1111%2Fj.1601-183X.2011.00742.x&data=05%7C02%7Cit4770%40princeton.edu%7C6a4bb1af54a14e9a729508dd7772226b%7C2ff601167431425db5af077d7791bda4%7C0%7C0%7C638798054677080617%7CUnknown%7CTWFpbGZsb3d8eyJFbXB0eU1hcGkiOnRydWUsIlYiOiIwLjAuMDAwMCIsIlAiOiJXaW4zMiIsIkFOIjoiTWFpbCIsIldUIjoyfQ%3D%3D%7C0%7C%7C%7C&sdata=oR1HlexS9amcO8FQ%2F%2BW87kqGhcyA5hdgOBGUrXUGicI%3D&reserved=0>, <https://nam12.safelinks.protection.outlook.com/?url=https%3A%2F%2Fdoi.org%2F10.1159%2F000360071&data=05%7C02%7Cit4770%40princeton.edu%7C6a4bb1af54a14e9a729508dd7772226b%7C2ff601167431425db5af077d7791bda4%7C0%7C0%7C638798054677093247%7CUnknown%7CTWFpbGZsb3d8eyJFbXB0eU1hcGkiOnRydWUsIlYiOiIwLjAuMDAwMCIsIlAiOiJXaW4zMiIsIkFOIjoiTWFpbCIsIldUIjoyfQ%3D%3D%7C0%7C%7C%7C&sdata=ts%2B4WQ0VbOMlyHxAb4kw%2Fy6u4YoYUVpNxqncLK0WBl0%3D&reserved=0>, <https://nam12.safelinks.protection.outlook.com/?url=https%3A%2F%2Fdoi.org%2F10.1098%2Frspb.2024.0121&data=05%7C02%7Cit4770%40princeton.edu%7C6a4bb1af54a14e9a729508dd7772226b%7C2ff601167431425db5af077d7791bda4%7C0%7C0%7C638798054677103054%7CUnknown%7CTWFpbGZsb3d8eyJFbXB0eU1hcGkiOnRydWUsIlYiOiIwLjAuMDAwMCIsIlAiOiJXaW4zMiIsIkFOIjoiTWFpbCIsIldUIjoyfQ%3D%3D%7C0%7C%7C%7C&sdata=julzl3FM3hlVOZZcMpWCy8buvN8B45inpAWnHjrDcjs%3D&reserved=0)>.

We also are excited about this result, and we thank the Reviewer for this positive comment and these new references! They have been incorporated in a larger discussion of *nlg2* conservation in Lines 628-642 of the Discussion, which dovetail with a request from Reviewer 1.

We also agree that a full phylogenetic analysis is out of scope, but now present results regarding genetic similarities to the fruit fly and human neuroligin gene models. *Nlg2* was identified as a likely ortholog with a robust, one-to-one reciprocal best hit (RBH) BLAST between honey bees and fruit flies, which is a highly stringent means of comparing sequences across species. The RBH-BLAST method (which is not specific to *nlg2*) was published in Traniello et al. 2019, and we cite and reference it in the Methods, but we now think it best to also add the specific method and reference in Results (Line 426) for clarity. For comparison to humans, we performed a conditional RBH-BLAST (cRBH-BLAST), which is more appropriate for distantly related species, and did not identify *nlg2* orthology. However, a straightforward peptide BLAST indicated that honey bee *nlg2* indeed shares strong structural similarities with human neuroligins, including *nlg2*. While percent identity is capped around 30%, similarity is strong enough to produce very low e-values (~10^-60^ and below). We are grateful to this Reviewer for the suggestion, and we now present these results in Lines 432-436 of the Results section.

2) Did the authors look at other "sociability" candidate (from work in bees, Drosophila, and/or vertebrates) genes in their transcriptomic data? It would be interesting to see whether other candidates show intriguing patterns similar to neuroligin.

We did! And we now reference these other findings in Lines 526-539 of the Results, along with the aforementioned addition to the Discussion (Lines 628-642).

3) In the Discussion, can the authors speculate what neuroligin might be doing in honey bees to drive aspects of social behavior?

Yes, this is an excellent idea for driving home the idea that variation in just a single gene rarely results in variation in social behavior, but some genes may play outsized roles in modulating behavioral state and internal motivation. This is an important point and is discussed in Lines 636-642 of the Discussion.

Specific comments:

- Line 122: Should " instrumentally" be replaced with "artificially" or similar?

We have changed “instrumentally” to “artificially” – both are correct, but the latter is a more accessible term (Line 137).

- Lines 130ff: Sample sizes need to be clearly stated.

They are now clearly stated in Lines 147-152.

- Lines 149ff: This automated detection of trophallaxis events is cool, but I am wondering whether this has been compared to manually scored behavior for some of the videos. In other words, how do the authors know that trophallaxis events involve two bees 1.7 mm - 7.4 mm apart and lasting between <3 seconds or >3 seconds?

Yes, comparisons to manually scored behavior have been made! Along with a similar request from Reviewer 1, we now add substantial text to Lines 174-187 of the Methods, clarifying how and when manual observations have been performed to assess detection rate and ground-truth the CNN.

- Line 306: 13,000 paired-end reads/sample seems rather low? How many genes?

We thank the Reviewer for catching this. There were 13,000,000 reads or 26,000,000 read-pairs, not 13,000. This has been corrected, and we apologize for the typographical error. We now present the corrected read number (Line 354).

- Lines 316ff: How many genes were analyzed? What filter criteria (i.e., above which TPM threshold in what portion of samples) were applied? How was the data normalized?

We agree that these details are very much lacking! We have added a new paragraph to the Methods (Lines 364-369) where our post-alignment processing and DESeq2 analysis is properly discussed.

- Line 367: How was "robust one-to-one orthology" confirmed? I assume by reciprocal BLAST? In Drosophila, both nlg-2 and nlg-3 have been implicated in aspects of social behavior. While beyond the scope of the current study, long-term, it will be good to assemble a phylogeny for this gene family.

This is correct – we used a RBH-BLAST between honey bees and fruit flies, and then performed a conditional RBH-BLAST between honey bees in humans, as discussed above. We now incorporate these findings in Lines 426-436 of the Results and more generally clarify it where mentioned in the text.

- Figure 5: The micrographs are very pixelated, which makes it difficult to assess these results.

We think this is related to the original formatting of the manuscript and have now supplied high-quality .tiff files for each figure.

Reviewer #3:

In this manuscript, the authors used a combination of automated behavioral tracking, GWAS, brain transcriptomic analyses, and FISH analysis to suggest that the trophallaxis frequency in honey bees correlates with mutations and expression levels of Nlg2, which is related to human sociability, suggesting the possibility that there is a common molecular neural basis for honey bee and human sociability. The notion that trophallaxis could be an indicator of honey bee "sociability" is novel, and the large part of the experiments aimed at identifying GVAS and gene expression variation correlated with trophallaxis frequency are well designed. On the other hand, the experimental evidence supporting the notion that trophallaxis frequency could be an indicator of honey bee sociability seems still not so much robust and needs further substantiation. Therefore, the possibility that trophallaxis frequency may correlate with traits other than "sociability" needs be tested in this
manuscript as well, which will make the conclusions of this paper more objective and convincing. My comments are as follows.

Major comments:

1.      Besides "sociability", the trophallaxis frequency in worker honey bees may also reflect the physiological status of individual worker, as stated in the Introduction section (Line 35). Since the fluid exchanged by trophallaxis in nurse bees is royal jelly secreted from the head glands (Crailsheim 1998), trophallaxis frequency may reflect the individual differences in the amount of royal jelly produced in nurse bees. (If some workers have low royal jelly production due to undeveloped head glands, would they still perform frequent trophallaxis?) To test this possibility, the authors need to test if there is any correlation between the development of the head glands (or the expression levels of royal jelly protein gene) with trophallaxis frequency (if the authors' notion is correct, we would expect no significant correlation). Alternatively, if the tendency of some workers to show high or low trophallaxis frequency does not change throughout their life (from nurse nees to
foragers), this may also suggest that trophallaxis frequency does not mainly reflect a physiological state but "sociability (by nervous system)" of the bees.

If it is difficult to perform this experiment, then the Discussion should clearly state that this alternative possibility is not tested, and the entire manuscript needs to be rewritten to take into account the possible contribution of individual differences in physiological status as well as "sociability (by the nervous system)" to the trophallaxis frequency. Currently, the manuscript is written on the premise that trophallaxis frequency is an indicator of "sociability" and needs to be toned down significantly.

This is an interesting set of ideas! First, we note that we do find *major royal jelly protein 6 precursor* in our list of genes positively correlated with trophallaxis, whereas several MRJP genes are negatively associated with locomotion. This is now included in our Results (Lines 535-537) and a new paragraph in the Discussion (Lines 700-711), because it supports the Reviewer’s suspicion that nutritional physiology plays a role in trophallaxis frequency but not locomotor state. This is important to address, and we appreciate the suggestion!

Unfortunately, bees are flash-frozen to preserve RNA and capture the most behaviorally relevant transcriptomic profiles possible, and fresh dissections are not possible for hundreds of bees undergoing collection in short period of time (i.e., within a time window of just a few hours). While flash-freezing is ideal for molecular biology, it distorts the morphology of the hypopharyngeal glands. We submerge our samples in RNA-laterICE before dissecting the mushroom bodies, but we’ve found that this also makes the head glands hard to assess. We briefly address this technical limitation in Lines 700-705 of the Discussion, but we agree with the Reviewer that head gland development, especially in light of the MRJP findings, would be exciting to determine in a future study designed for that purpose.

2.      For Figs. 2 and 3; when the authors obtained the results that individuals from colony R41 are homozygous for the Nlg2 allele, while individuals from colonies R2 and R8 are predominantly heterozygous, they should have performed a similar analysis for wild type individuals derived from colonies R2, R8 or other colonies. I assume that the authors have this data, so I would like to strongly suggest the authors to reanalyze the data and include the results in this manuscript.

Single-drone insemination (SDI), in which queens are artificially inseminated with semen from a single male, increases the precision of molecular analyses by reducing intracolony heterogeneity, but has no bearing on whether the bees are wild-type or not. In fact, the bees used in this study all were wild-type. We see there is need to clarify the rationale for using SDI queens in this study and to emphasize that the bees were wild-type, and we do so in Lines 139-141 of the Methods. This is an important clarification and we thank the Reviewer for bringing up the issue.

Minor comments:

1.      For the reasons stated in my Major comment 1, the term "trophallaxis sociability" should be replaced with a term "trophallaxis frequency" for the entire manuscript.

We agree that the physiological and social dimensions of trophallaxis must be further described. “Trophallaxis frequency” is a simpler and more descriptive term, and we have switched it in the Methods and Results. We also think it best to retain “trophallaxis sociability” in key places to emphasize the higher-level themes of the paper. Although knowledge about the physiology of trophallaxis is incomplete, it is reasonable to assume that trophallaxis, as a social behavior performed intensively by many colony members that involves exchange of nutrition and signaling molecules, emerges from a combination of external (e.g., colony needs) and internal (e.g., physiological state, hunger, fullness) factors, like other forms of social behavior. We discuss this more in-depth in Lines 45-50 of the Introduction, and we hope that we have better justified a more limited use of this term.

We note that this was labeled a “minor” comment, and we are happy to further clarify our position in the text as the Reviewer sees fit.

2.      Please consider showing the mutations in the intron of the Nlg2 gene in a supplementary Figure and discuss the possible mechanism by which the mutations alter the expression levels of Nlg2.

We discuss possible mechanisms of altered *nlg2* expression in Lines 609-612 of the Discussion.

3.      In FISH experiments, Nlg2 is also strongly expressed in inner-compact KCs and optic lobes. Please describe the presumed function of Nlg2 in these neurons.

This is an excellent idea and we have some history with exploring these KCs in the context of social behavior via *in situ* hybridization (in Traniello et al. 2019). We now include this discussion in Lines 663-677 of the Discussion.

4.      The values on the left vertical axis in Fig. 4a are all -4?

Yes, this is not an error: this particular GO analysis software uses the highest and lowest p-values to make the color scale (for example, 1.0 x 10^-4^ and 5 x 10^-4^ occupy opposite ends of the color scale). We now clarify this in the Figure 4 legend and thank the Reviewer for this keen observation!


Reviewer #4:

The interesting manuscript by Traniello et al. investigates individual differences in honey bee social behavior and their underlying molecular correlates. The authors identify genetic and molecular correlates underlying trophallaxis in honey bees, a highly social insect model by combining genome sequencing and brain transcriptomics with behavioral tracking. Honey bees which were "socially unresponsive" had shorter and briefer trophallaxis interactions compared to age-matched individuals which were more responsive to social stimuli in an earlier study. The authors of the current study reveal 18 SNPs which are associated with some variation in social behavior. Some of these are localized to genes associated with social behavior in other species and the authors suggest fundamental and shared molecular mechanisms underlying sociability. The identification of two nlg2 SNPs associated with variation in trophallaxis is particularly exciting, because these genes have been associated
with human autism. The link between nlg2, reduced motor synchrony between individual honey bees and human autism appears less convincing, though. Overall, this study reveals exciting new insight into the molecular mechanisms underlying social behavior, although, naturally, honey bees are evolutionarily very distant from humans.

The manuscript is very well written. I only have a couple of minor points to consider.\

We thank the Reviewer for the kind words! We hope our responses are satisfactory and improve the Reviewer’s interpretation of the manuscript.


Minors
1)Line 46
There are other factors impacting task performance in honey bees, please add physiology and hormones here and add appropriate citations (maybe including some outside the Robinson group)

This is an excellent idea and we now include a number of references in the Introduction, specifically in Lines 44-50. The new material better helps set up division of labor and trophallaxis, which are unpacked later in the Introduction.

2) Line 146-153
What was the error date in the detection of bar-coded bees? How well can bees be distinguished if they are on top of each other?

Great question! We are pleased to report that this CNN-based tracking system features excellent detection metrics for trophallaxis,. We now reference this in the manuscript in Lines 174-187. While a bee cannot be identified if her tag is missing, the design of the observation hive (space between glass and honeycomb) makes it very difficult for bees to be on top of each other, so tags are rarely occluded by nestmates. A tag cannot be identified when a bee is cleaning a cell or feeding a larva, but she is also not performing trophallaxis at that time.

3)Lines 252
Why did you use the thorax for DNA sequencing and not the brain?
Did you pool samples for DNA sequencing?

We did not perform any pooling for DNA sequencing; each bee is analyzed individually. We used the thorax because it reliably generates large DNA yields and is not typically a tissue of interest for behavioral transcriptomics experiments in our lab. Because this study does not explore somatic mutation in the brain, we expect the genome in the thorax to generally be representative of individual genotype and we thus have the luxury of selecting a more DNA-rich tissue that allows us to save the brain for behavioral transcriptomic analyses.

We make this clearer in the Methods, in Lines 290-293.

4) Line 257ff
Did you pool mushroom bodies for RNAseq? If so, how did you pool?

We did not pool mushroom bodies for RNA-Seq, so each replicate represents a single bee. The mushroom bodies contain several hundred thousand cells and we are regularly able to extract up to a microgram of RNA from a single sample. We now clarify this in the Methods in Lines 306-309.

5) Why did you only study 13,000 reads of brain MBs? Is there a writing mistake and you mean 13,000,000? It is still low. Why did you not sequence deeper with a small amount of tissue and a comparatively low gene expression?

We thank this Reviewer and Reviewer 2 for catching this typo! Yes, the number was meant to be “13,000,000,” and we have updated this to report the number of read-pairs, which is closer to >26,000,000 per sample (Line 354).
