# Supplementary material for: Genetic variation influences food-sharing sociability in honey bees
Source: PLoS Biol. 2025 Sep 16;23(9):e3003367. doi: 10.1371/journal.pbio.3003367 (PMC12440162; doi:10.1371/journal.pbio.3003367)
Supplement: Attachment — Submitted filename: TranielloB2B_FinalRevision_R2R.docx [file pbio.3003367.s016.docx]

Reviewer #1:

[identifies himself as Tomas Kay]

I am greatly impressed by the changes that the authors have made in response to the first round of reviews. They have done an excellent job. All concerns have been thoroughly and thoughtfully addressed and I am excited to see this manuscript published.

All our gratitude to Dr. Kay for the comments that greatly improved the manuscript.

Reviewer #3:

I appreciate that the authors took my comments seriously and attempted to improve the manuscript. However, I still find several points that I believe need further revision. My comments on the authors' responses to my previous comments are as follows.

Major comment 1:

I understand that the previous samples are unavailable. That is unavoidable. However, in that case, it is necessary to state in the Discussion section that "In this study, the degree of development of the hypopharyngeal glands in the samples was not investigated, and so the possibility that the degree of hypopharyngeal gland development influences trophallaxis frequency cannot be ruled out."

In the revised manuscript, lines 535-537 and 700-711 have been added to include the information that "the mushroom body gene list includes major royal jelly protein 6 precursor." However, while this may suggest a new function of major royal jelly protein 6 precursor in the mushroom body, it does not indicate the degree of development of the hypopharyngeal glands in the samples (at least, there is no evidence to support this). Therefore, while it is acceptable to add lines 535-537 and 700-711, it is also necessary to include the following statement in the Discussion section: "In this study, the degree of development of the hypopharyngeal glands in the samples was not investigated, and so the possibility that the degree of hypopharyngeal gland development influences trophallaxis frequency cannot be ruled out."

We appreciate the careful interpretation and have added the requested text to Lines 701-703 of the Discussion.

Major comment 2:

I understand the authors' explanation.

Minor comment 1:

I understand that the authors wish to use the term "trophallaxis sociability" in important sections to emphasize the main theme of the paper. However, I do not agree that this term should be used in the Introduction or Results section, as it should be used in the Discussion of this paper, where the results of this paper indicate that the Nlg2 gene is associated with trophallaxis frequency. While I appreciate that the authors have removed most instances of the term "trophallaxis frequency" from the Introduction and Results sections, two instances remain in the Introduction section and have not been removed from the figure legends. I believe it is necessary to remove these terms "trophallaxis frequency" from these portions first.

We have made the requested edits to the Figure titles, legends, axes (now corrected in Fig. 2a x-axis, 3b figure titles, 3d x-axsis, and Supp. Fig. S1 x-axis). We note that a new title was suggested for this manuscript, “Genetic variation influences food-sharing sociability in honey bees.” We wonder if this Reviewer will accept “food-sharing sociability” in three instances toward the end of the Introduction (Lines 109, 122, and 131).

On the other hand, for example, after adding the following sentence at the end of the fourth paragraph of the Discussion (line 605): "Based on the findings of this study, trophallaxis frequency may serve as a measure of an individual's sociability, and in that sense, trophallaxis frequency could potentially be rephrased as 'trophallaxis sociability.'" I believe it would be appropriate to use the term "trophallaxis sociability" in the subsequent sections of the Discussion.

We have added this line (with minor edits to fit the writing style, Lines 608-610).

Minor Comment 2:

The author misunderstood my request due to my poor explanation. I simply requested to explain the possible mechanisms by which mutations in the intron of Nlg2 could affect the transcription level of Nlg2. The description in lines 609-612 does not address my request, so please reconsider. Has the mutant sequence of the Nlg2 gene already been shown in the data?

We apologize for the misunderstanding. We added to those sentences to specifically deal with gene expression. For the second point, the mutant sequence of the Nlg2 gene was detected in the DNA sequencing, but we do not have mechanistic connections between this and gene expression, but we note that *nlg2* expression was indeed lower in the SNP-containing colony (Lines 603-608).

Minor comment 3:

I understand the authors' explanation.

Minor Comment 4:

I understand the authors' explanation.
